# State Board of Health

**Published:** 1877-01

**Authors:** 


### Editorial.

### STATE BOARD OF HEALTH.

A general supervision of the sanitary condition of the State, and the preparation of a perfect statistical record of diseases, births and deaths, seem to be the objects for which this Board was created. In their accomplishment the public interest will certainly be subserved, to a degree little expected by those who have not given the subject due consideration. In order to this, however, a perfect system must be afforded, particularly in regard to the statistical record. Calculations based upon partial or imperfect reports of diseases, births and deaths are necessarily erroneous, and instead of a blessing, entail upon the medical profession, and through it, upon the public, decidedly injurious results. For instance, the failure to report one birth, one death, or the disease from which death occurred, in each county, or even a dozen counties of the State, will cause such fatal error in the record of vital statistics and the character of prevailing disease, as not only to destroy their usefulness, but to make them a source of detriment to the people. It is worse than idle, then, to say that some good is effected even by imperfect statistical reports. Calculations founded on error are certainly worse than none at all.

Now, our duty as medical journalists leads to the inquiry, whether the Georgia Board of Health is accomplishing the objects—as set forth above—for which it was organized? From our standpoint, with the lights before us, we unhesitatingly express the opinion that it is not. The correctness of this does not necessarily involve a want of competency or fidelity in the discharge of duties incumbent upon members of the Board, but the failure is attributable mainly to palpable defects in the State law under which they were organized. It may be true that the framers of the bill in our State Legislature may not have availed themselves of the experience of those entrusted with the conduct of similar boards in other States; but it must be remembered that a test under all the peculiar circumstances which may surround a particular plan, is the only guide to success.

The usefulness of such organizations depends, as we have said, upon the perfection of statistical reports, and in the act creating this board it was sought to compel returns of births, deaths, etc., by means which, when put to the test, have utterly failed. The law makes it the duty of physicians to report to the Board all the births, deaths, etc., that occur in their practice. A penalty of ten dollars was at first required for failure to comply with this provision of the act. This was repealed at the last session of the Legislature, so that with and without this alleged objectionable feature, the law fails to be complied with —some making partial returns, others none at all. In addition to this requirement of physicians, it is enjoined upon the Ordinary of each county to aid in securing such returns, and yet the statistical reports which the Secretary of the Board is able to make are imperfect, and therefore amount to nothing.

That a material change in the provisions of the act creating the Board is demanded, no one can question, but exactly what that change should be is more difficult to dicide. Should the tax oath be made to include the items of births, deaths, and nature of the fatal diseases occurring in the family since the previous tax return, an approach to reliable reports would be had. Even with this plan, however, statistics must of necessity be sadly defective, on account of the considerable number of families paying no tax, while tax payers, not a few, fail to make returns to the receiver.

Similar boards, in other States, have been in operation for some time, and are pronounced successful. Our information is that some of them insure compliance with the law by pecuniary penalties, but so far as tested in this State, the plan was found not particularly encouraging. We presume some plan will be matured by the Board to be acted on at the ensuing session of the Legislature, by which at least a near approach to success may be attained.

